# Case of Contracted Œsophagus

**Published:** 1803-06-01

**Authors:** 

**Affiliations:** Limehouse


### Case *of* Contracted Œsophagus; *communicated by* Mr. Smith, *Jun. of Limehouse.*

On the 14th of April last, I was desired to visit Mrs. Elizabeth Brown of this neighbourhood, who laboured under what appeared to be a contraction of the œsophagus; she could swallow no solid food, and was also much affected with dyspnœa, particularly on attempting to lie down, being, when in bed, obliged to have the trunk elevated to nearly an upright position by means of pillows. On enquiry, I found she had felt a little impediment to swallowing near a twelvemonth back, and in January last was attacked by a severe catarrhal affection, but did not find the difficulty of swallowing increase till about seven weeks ago, when it became much worse, and the difficulty of breathing also was at times very great, occasioning much distress and alarm to the patient and her friends; she feeling a lump, to use her own expression, in her throat, which obstructed her swallowing, and appeared as if it would strangle her. I desired her to take some liquid in my presence; which she did, but could swallow very little at a time, and with a peculiar kind of noise, not unlike a person in the act of gargling the throat. I conjectured there must be a contraction of the œsophagus, and that there must be some tumour which pressed upon the trachea and occasioned the difficulty of respiration, particularly as the throat appeared rather swelled, just below the cricoid cartilage. I had a blister applied round the throat, and one to the upper part of the sternum, and gave her a grain of calomel night and morning, which she took in a little gruel.

gruel. The blisters were kept open by the ung. Sabinæ, and discharged greatly. Two days after she thought herself relieved, and swallowed a small piece of cake soaked in tea, or milk and water, but on the following day the symptoms were more severe than ever. I wished to introduce a bougie into the œsophagus, to endeavour to dilate the stricture, but could not prevail on her to consent. She continued in this state, often expressing an inclination for solid food, but unable to take it, till Friday the 22d at night, when the difficulty of breathing became more violent, and, on Sunday, the 24th, about noon, excessive, when she expired, apparently suffocated. Her throat appeared rather more swelled a day or two before her death, but in a few hours after it wholly subsided. Permission being obtained to open the body, the following were the appearances which presented: The stomach was much contracted, and the rugæ on its internal surface very large and numerous, which is to be attributed to the small quantity and nature of the aliment received into it for some time past. The liver appeared to have been at some former period slightly inflamed, as broad, irregular patches of a buffy appearance were found on its surface, not however extending far into its substance, which on cutting into it presented a healthy appearance. There was a slight adhesion of the right lung to the pleura. On cutting into the trachea, a considerable quantity of pus issued out, which had made its way into it at its upper and back part, where it is not cartilaginous, at which part it rather projected in the form of a small tumour, with an apex a quarter of an inch long, which was found to communicate with the œsophagus. The parietes of this latter were very much thickened at this part, and the passage was, for the length of three inches, so contracted as to be impervious to a crow quill. It also contained a quantity of matter not unlike to curdled milk. The inferior part of the œsophagus was not altered in its structure, and all the other viscera were perfectly sound. The pus found in the trachea had flowed through the aperture above mentioned. It plainly appears, the death of the patient was caused by the bursting of this abscess into the trachea, and all the preceding symptoms of dyspnœa by the pressure of the tumour at that part. The subsiding of the tumour about or rather after the time of her death, is accounted for by the evacuation of the pus into the trachea. The appearance of the pus was very similar to that found in scrofulous abscesses.

Inflammation might perhaps at first take place in that part of the œsophagus next to the trachea, which might extend to the membranous part of the trachea itself, and lay the foundation for this distressing disease, which for several months appeared to make no progress, and was slight in its symptoms, when it suddenly became very much aggravated, and at length put a period to the life of the patient.

